# Waveguide-Based Biosensors for Pathogen Detection

**DOI:** 10.3390/s90705783

**Published:** 2009-07-21

**Authors:** Harshini Mukundan, Aaron S. Anderson, W. Kevin Grace, Karen M. Grace, Nile Hartman, Jennifer S. Martinez, Basil I. Swanson

**Affiliations:** 1 Physical Chemistry and Applied spectroscopy, Chemistry Division, Los Alamos National Laboratory, Los Alamos, New Mexico 87545, USA; 2 Integrated Space Research-4, Los Alamos National Laboratory, Los Alamos, New Mexico 87545, USA; 3 nGimat™, 5315, Peachtree Industrial Blvd., Atlanta, GA30341, USA; 4 Centers for Integrated Nanotechnology, Los Alamos National Laboratory, Los Alamos, New Mexico 87545, USA

**Keywords:** planar optical waveguides, biosensors, thin film, fluorescence, immunoassay, pathogen sensor

## Abstract

Optical phenomena such as fluorescence, phosphorescence, polarization, interference and non-linearity have been extensively used for biosensing applications. Optical waveguides (both planar and fiber-optic) are comprised of a material with high permittivity/high refractive index surrounded on all sides by materials with lower refractive indices, such as a substrate and the media to be sensed. This arrangement allows coupled light to propagate through the high refractive index waveguide by total internal reflection and generates an electromagnetic wave—the evanescent field—whose amplitude decreases exponentially as the distance from the surface increases. Excitation of fluorophores within the evanescent wave allows for sensitive detection while minimizing background fluorescence from complex, “dirty” biological samples. In this review, we will describe the basic principles, advantages and disadvantages of planar optical waveguide-based biodetection technologies. This discussion will include already commercialized technologies (e.g., Corning’s EPIC^®^ Ô, SRU Biosystems’ BIND^™^, Zeptosense^®^, etc.) and new technologies that are under research and development. We will also review differing assay approaches for the detection of various biomolecules, as well as the thin-film coatings that are often required for waveguide functionalization and effective detection. Finally, we will discuss reverse-symmetry waveguides, resonant waveguide grating sensors and metal-clad leaky waveguides as alternative signal transducers in optical biosensing.

## 1. Introduction

Light is reflected when traveling through a boundary between materials with different refractive indices (refractive index of materials A > B). At a critical angle of incidence, the light is reflected with near perfection, producing total internal reflection (TIR), a simple physical phenomena with important practical consequences for biosensing. Planar optical waveguides are comprised of an optically transparent guiding layer with a refractive index that is higher than the substrate layers. With careful selection of the guiding material, and gratings for incoupling of light, biosensors based on planar optical waveguides can provide versatile and robust transduction sensor platforms for the rapid and sensitive analysis of complex environmental and medical samples. Planar waveguide geometries also allow for facile integration with sample delivery and detection systems, and for the functionalization and patterning of arrays of recognition elements onto the surface, allowing for simultaneous detection of multiple analytes using a single waveguide transducer [1,2].

When excitation light is coupled into the guiding layer of a planar optical waveguide, light is guided over long distances by TIR. Although most of the light is confined within the guiding layer, a small portion (the evanescent field) extends out into the substrate and into the medium (the biological sample). This evanescent field falls off exponentially as the distance from the waveguide surface increases, and is effectively zero at a distance less than one-half the wavelength of the coupled light. Thus sensitivity is highly enhanced because of the large degree of discrimination between surface bound molecules and contaminants within the sample solution. Evanescent field sensing can be applied to several different transduction approaches including evanescent fluorescence detection, monitoring of refractive index changes or detecting spectroscopic shifts. The current manuscript will largely focus on fluorescence-based detection platforms, with brief discussions on other transduction approaches.

Waveguide sensor systems have been the subject of a large number of investigations over the last two decades. The concept of evanescent field sensing was initially reported by Lukosz and Tiefenthaler in 1983 [3,4]. While using thin, high refractive index SiO_2_-TiO_2_, waveguides with incoupling gratings, they discovered variations in incoupling angles due to changes in the effective refractive index of the guided modes due to variations in humidity. Subsequently, these authors proposed and demonstrated application of this observed effect toward chemical, and biochemical sensing [3]. Evanescent field sensing is now well established and sensor systems based on both single mode and multimode waveguide structures have been developed and demonstrated by numerous investigators. The physical properties of planar optical waveguides that make them ideal for biosensing applications are discussed in detail below (Section 3). Examples of commercialized technologies as well as new technologies under development are discussed in Section 7.

There are two major classifications of waveguide systems- multimode and single mode. Multimode waveguides have a thickness much greater than the wavelength of the excitation light and are typically fabricated using glass, polymer or silica materials making them relatively inexpensive and easy to manufacture. The thickness of the waveguide is on the order of several microns allowing simple edge coupling of excitation light into the waveguide structure. Unfortunately, due to relatively low levels of signal intensity, detection methods for florescence-based, multimode sensors, typically rely on Peltier-cooled CCD cameras.

Single mode waveguides are typically comprised of a very thin (< wavelength of excitation) high dielectric index film (e.g., silicon oxynitride, tantalum pentoxide and others) deposited on a low index substrate [5]. The films are typically fabricated using thin film deposition techniques. Alternative fabrication approaches include the use of sol gels [6] and ion deposition methods. Details on thin film, single mode, planar waveguide design and fabrication are presented in Section 2. Single mode waveguides with large index differences between the film and substrate (high contrast waveguide systems) offer greater sensitivity due to the high field intensity at the surface. Additionally, single mode planar waveguide supports several thousand reflections per centimeter of beam propagation for visible wavelengths, two orders of magnitude higher than multimode planar and fiber waveguides. Finally, single mode planar waveguides with high contrast enable the rapid decay of the evanescent field away from the waveguide surface with no appreciable intensity beyond one-half the wavelength of the excitation light (∼250–300 nm), while low contrast multi-mode configurations typically exhibit a penetration depth of 1–2 microns. As a result, the strong spatial filtering effect further enhances sensitivity by minimizing background from interferents and allows direct analysis of complex samples while eliminating the need for additional rinsing and drying steps. The requirements for highly sensitive detectors are also decreased allowing for use of room temperature based CCD cameras or miniature fiber optic spectrometer systems. However, this increased sensitivity for single mode waveguides requires certain modifications of the waveguide such as thin film deposition and use of grating couplers to couple excitation light into the waveguide films. Another potential problem includes photo-bleaching effects when fluorescent organic dyes are employed for detection due to the strong field intensity at the waveguide surface. However, this problem can be minimized or eliminated by the use of photostable quantum dots (QDs) as fluorescence reporters. Exploiting the broad Stokes shift of these nanoparticles allows for the simultaneous excitation of multiple QDs at a single wavelength, which can be exploited for multiplex detection of analytes on waveguide-based platforms (Section 6).

Incoupling of light into waveguides can be achieved by end-fire coupling or with the use of gratings. Because single-mode waveguide films tend to be thin, on the order of 0.1μm for devices with high sensitivity, end fire coupling is impractical. While this technique works well with waveguides having large core waveguide dimensions, it requires frequent change of the sensor wafer precluding its use in practical applications. This change is necessitated by a requirement of precise XYZ alignment tolerances for efficient coupling and end face preparation of single mode waveguides. The use of grating couplers will be discussed in Section 2.

In this review, we will present the basic physical and optical principles of single mode planar optical waveguides that make them suitable for biosensing applications. We will review the current methods for waveguide-surface functionalization to make them compatible for biological assays, while discussing the different biological sensing applications for which they have been used. We will discuss a waveguide-based biosensor developed by our team at the Los Alamos National Laboratory (LANL) and the adaptation of this technology to biosensing applications in single and multiplex formats. We will also present some commercial applications of waveguides in biosensing. It is impossible to incorporate detailed description of all waveguide-based sensing technologies into a single review article and yet, do justice to them. Therefore, this review article will limit itself to the described scope. Some novel technologies such as silicon photonic biosensors, that have made tremendous impact on such applications will only be briefly discussed, but have been reviewed in detail elsewhere [7].

## 2. Waveguide Design and Fabrication

### 2.1. Waveguiding Principles

Planar optical waveguides are based on a thin, optically transparent film with a refractive index that is higher than adjacent substrate and superstrate mediums. Under this condition, light coupled into the thin waveguide film will be confined to that layer and can propagate over useful distances. Furthermore, the planar waveguide configuration offers important advantages as optical elements may be integrated into or onto the waveguide film surface to form integrated optical circuits, which are thin film analogs of bulk optical circuits [8]. The telecommunication industry quickly adopted the planar waveguide optical technology due to emerging demands for greater bandwidth and freedom from electromagnetic interference. Similarly, in the early 1980s, the potential for sensing physical and chemical or biological parameters was recognized, resulting in rapid surge in interest in the sensing arena. Successful application of the planar optical waveguide technology is, however, strongly dependent on proper waveguide design. In the following sections waveguide operation and fabrication are discussed and designs for optimized waveguide structures are then presented in later sections (Sections 3 and 6).

**Figure 1.**
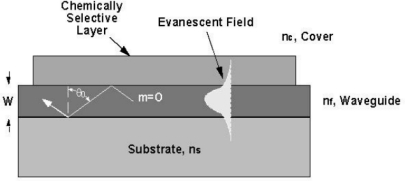
Waveguide cross-section illustrating zig-zag ray model for optical propagation.

For modeling and analysis purposes, an optical waveguide may be described quantitatively using a dispersion relationship incorporating the step index waveguide parameters n_f_ (film index), n_c_ (cover film index), n_s_ (substrate index), and W (waveguide film thickness) [9]. A convenient model for describing waveguiding and defining it’s sensing capability is based on a “zig-zag” optical ray model for the reflection of an optical ray between the two dielectric waveguide surface boundaries as shown in Figure 1. The dispersion relationship is defined by the “transverse resonance condition”, which requires the sum of all phase shifts perpendicular to the direction of propagation in the waveguide to be a multiple of 2π̣ (2mπ, where m = 0, 1, 2,...) for one “zig-zag” period of the light propagating within the waveguide. Thus, the total phase shift associated with the transverse motion between the two boundaries must be an integer multiple of 2π for each full cycle. For one transverse passage through the waveguide, a phase shift of kn_f_Wcosθ occurs (k = 2π/λ). One full period, however, requires two transverse passages. Additionally, phase shifts of −2γ_c_ and −2γ_s_ occur due to total internal reflection at the cover and substrate boundaries of the waveguide. Thus an equation of the following form results:

(1) $$\left(2{\text{kn}}_{f}\;W\text{cos}\theta \right)-2\gamma_{c}-2\gamma_{s}=m(2\pi )$$

(2) $$\text{where}:\;\;\;\;\;\;\;\;\;\;\;\;\;\;\;\;\;\;\;\;\;\;\;\;\;\;\;\;\;\;\;\;\;\;\;\;\gamma_{c}={\text{tan}}^{-1}\left[{\left(n_{f}\;{}^{2}\;{\text{sin}}^{2}\;\theta -n_{c}{}^{2}\right)}^{1/2}/\left(n_{f}\text{cos}\theta \right)\right]$$

(3) $$\text{and}:\;\;\;\;\;\;\;\;\;\;\;\;\;\;\;\;\;\;\;\;\;\;\;\;\;\;\;\;\;\;\;\;\;\;\;\;\;\;\;\;\;\;\gamma_{s}={\text{tan}}^{-1}\left[{\left(n_{f}{}^{2}{\text{sin}}^{2}\theta -n_{s}{}^{2}\right)}^{1/2}/\left(n_{f}\text{cos}\theta \right)\right]$$

Because the waveguide is thin and the index differences are small, waveguiding occurs only at discrete values of θ with each discrete angle corresponding to a propagating mode. Waveguides with propagation at only one specific value of θ are referred to as single mode waveguides while those with propagation at more than one value of θ are described as multimode.

The term n_f_ Sinθ is defined as the “effective mode index” (N_eff_) and represents an effective refractive index that is characteristic of the waveguide material system and configuration. The basis for this definition is illustrated graphically in the plot of the electric field distribution of a single mode waveguide shown in Figure 1. Associated with a guided optical wave is an exponentially decaying electric field distribution, the evanescent field, extending into the cover and substrate medium. The 1/e penetration depth of the evanescent electric field into the substrate (W_s_) and cover medium (W_c_) may be defined using the following equations:

(4) $$\text{Substrate}:\;\;\;\;\;\;\;\;\;\;\;\;\;\;\;\;\;\;\;\;\;\;\;\;\;\;\;\;\;\;\;\;\;\;\;\;\;\;\;\;\;\;\;\;\;\;\;W_{s}=1/(2/\pi ){\left(n_{f}{}^{2}\;{\text{sin}}^{2}\theta -n_{s}{}^{2}\right)}^{1/2}$$

(5) $$\text{Cover}:\;\;\;\;\;\;\;\;\;\;\;\;\;\;\;\;\;\;\;\;\;\;\;\;\;\;\;\;\;\;\;\;\;\;\;\;\;\;\;\;\;\;\;\;\;\;\;\;\;\;\;\;\;W_{c}=1/(2/\pi ){\left(n_{f}{}^{2}\;{\text{sin}}^{2}\theta -n_{c}{}^{2}\right)}^{1/2}$$

It is the interaction of the evanescent field with the cover medium that defines the basis for guided wave optical sensing of biological agents binding to the surface of an optical waveguide. The actual transduction step may rely on: (a) direct detection of a fluorescence signal induced at the surface of a waveguide by the evanescent wave, (b) direct detection of refractometric changes (interferometric) occurring on the surface of the optical waveguide or alternatively (c) by monitoring guided wave attenuation due to absorption or scattering (spectroscopic).

The above equations describe the propagation of a transverse-electric (TE) polarized guided wave with its electric field vector parallel to the surface of the planar waveguide. Similar equations can be developed for TM polarization where the electric field vector is perpendicular to the planar waveguide surface. Although TM polarization offers a slight advantage in terms of overall detection sensitivity, it is susceptible to strong polarization conversion (TM to TE) within a waveguide and can limit input coupling geometries when used with grating input couplers. Conversely, TE polarization is much less susceptible to those limitations.

The overall sensitivity of an optical waveguide system for selective detection of a biological molecule or intact agent binding to a waveguide surface depends strongly on the waveguide material and design. Specifically, optimizing the overlap of the exponentially decaying electric field with the cover film and maximizing the electric field strength at the waveguide surface defines detection sensitivity. In practice, a thin, high refractive index waveguide film deposited on a low index substrate leads to optimum detection sensitivity.

### 2.2. Planar Waveguide Material Systems

To perform satisfactorily in a sensor system, the waveguide material systems and deposited films must meet fairly stringent requirements. The key elements of the waveguide material system include the substrate, waveguide film, and any deposited thin film materials (the sensing film) that may be used for signal transduction based on molecular recognition. The actual selective binding chemistry is more or less defined by the application and is not discussed in this section although it must required to exhibit low optical attenuation at the wavelengths of interest.

The substrate material choice is critical as optically transparency and optically smooth surfaces are essential for good waveguide performance. For optimum sensitivity, a low index substrate is preferred and glassy type materials with refractive indices centered ∼ n = 1.5 typically performs well in this regard. In practice, fused silica (SiO_2_) represents an excellent substrate material with a nominal refractive index of 1.457 in the visible portion of the optical spectrum. Furthermore it is easily polished to achieve a 10^−5^ scratch-dig specification and an rms surface roughness of 0.5 nm or less. These specifications have been found to reliably produce high quality waveguide operation over the visible spectrum. Silicon wafers with thermally grown SiO_2_ may also be used as substrates. Fused silica offers an additional advantage in that it is easily etched by wet or dry processes. In contrast, other high-grade optical glasses such as BK-7 are based on a mixture of metal oxides that etch at differing rates, resulting in rough etched surfaces and contributing to increased levels of optical scattering.

Deposited waveguide films also must meet the same stringent requirements noted above and suitable waveguide materials systems include silicon oxynitride (SiO_x_N_y_, n = 1.6 to 2.0), tantalum pentoxide (Ta_2_O_5_, n = 2.1 to 2.3), and titanium dioxide (TiO_2_, n = 2.5 to 2.8). These material systems produce low loss waveguides with attenuations typically in the range of 0.5 to < 2.0 dB/cm, more than adequate for most sensor applications [5]. Single crystal substrates such a lithium niobate (LiNbO_3_) have also been used for sensor-based waveguides, primarily to take advantage of their electro-optic properties and application to signal processing. However the high refractive index characteristic of these substrate materials limits their potential detection sensitivity. Deposition methods for thin film waveguides are outlined in Table 1 [10] and all have been used for waveguide film deposition. Of these methods, Ion Beam Sputtering (IBS) provides outstanding results. IBS deposited films offer excellent film adhesion, ultra dense coatings with refractive indices approaching those of bulk materials, and deposited film roughness of 0.5 Å when deposited on a substrate of with equal or better smoothness. This is in contrast to conventional deposition techniques producing surface roughness of 10 Å or greater and ion assisted techniques producing 4 Å rms roughness [11].

### 2.3. Optical Input/Output Coupling Approaches

As mentioned in the introduction, a practical sensor system should be able to couple light in and out of a waveguide system with relative ease and high efficiency. nGimat^™^ and Los Alamos National Laboratory have successfully used grating couplers for this process [12]. Grating couplers exhibit greatly relaxed positional alignment tolerances, of the order of ± 50 μm *versus* ≤ 1 μm for end-fire coupling and angular alignment tolerances of the order of ± 1.5°. Additionally, coupling efficiencies of 25 to 40% are easily realized and input coupling may occur from either surface of the waveguide. The input/output coupling geometry is flexible, with coupling possible over and angular range of + 45° to −20° with respect to the waveguide surface normal.

**Figure 2.**
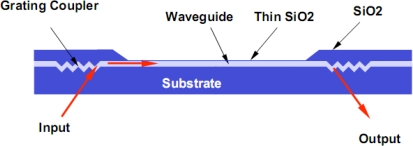
Grating coupler for efficient input/output.

Grating coupler fabrication relies on standard photolithographic techniques and a reactive ion etching process. The grating configuration used at LANL and nGimat^™^ is illustrated in Figure 2. The grating pattern is generated using a holographic interference technique. The developed photoresist pattern is etched into the substrate and the waveguide film is then deposited onto the substrate. This approach reduces the tight tolerances on grating etch depth encountered when etching the grating pattern directly into the waveguide film. After waveguide film deposition, the grating region is overcoated with a 1 μm thick IBS deposited SiO_2_ film. This physically protects the grating and, most importantly, isolates it from ambient environmental effects that might introduce detuning of the coupling angle. Thus the medium over the sensing surface may change from air to water with no change in coupling angle. Note the importance of utilizing a dense, non-porous film be utilized as the overcoat as water infiltration that would cause a slow detuning of the input-coupling angle. The same thick dense SiO_2_ overcoat also defines the sensing and reference channel regions.

### 2.4. Planar Waveguides and Integrated Optic Circuits

As noted previously, the planar waveguide configuration lends itself to integrated optic circuits where thin film versions of bulk optical systems may be implemented. In particular, LANL and nGimat™ have developed multi-channel waveguide sensor configurations incorporating not only grating input/output couplers but also other elements such as thin film mirrors and beam combining elements. The integrated optic circuits (IOC) have been applied to both fluorescence and interferometric sensing applications.

**Figure 3.**
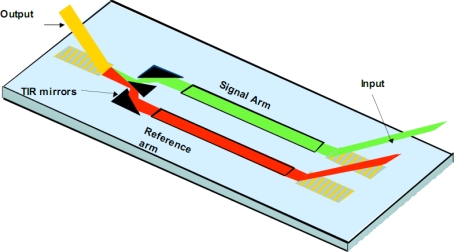
Physical Configuration for efficient input/output coupling.

For discussion purposes, a interferometric sensor is illustrated in Figure 3. We have developed single and multi-channel (n = 4) variants of the same. Key IOC elements include grating couplers, the waveguide film with patterned sensing and reference arms, and a beam combiner. The interferometric sensor functions by directly detecting the selective binding of a biological agent onto a waveguide surface. The binding step causes a change in N_eff_ that is easily detected by interfering the guided wave traversing the signal arm of the interferometer with a guided wave traversing the reference arm. By monitoring the resulting interference pattern, any phase delay due to a change in the signal arm is easily detected. The phase delay is quantitatively related to the amount of biological agent bound to the waveguide surface and correspondingly the agent concentration in the sample solution undergoing testing. By treating the reference arm of the interferometer to look biologically similar to the signal arm, non-specific binding may also be minimized [13]. Interferometric sensor systems have been used for chemical kinetic studies as well as detection of hazardous biological agents including toxins, bacteria and viruses.

## 3. Optical and Physical Principles of Waveguide-Based Detection

Planar waveguide sensing methodologies rely on the overlap of the evanescent field associated with a guided optical wave and the cover medium on or above the waveguide surface. This applies to both fluorescence and the interferometric sensing approaches. For fluorescence applications, the intensity of the guided wave excitation must be optimized at the boundary layer where capture of the biological target is concentrated, although the extent of the evanescent field must be restricted so as to limit fluorescence due to other materials present in the medium undergoing testing. Similarly, for interferometric sensing the evanescent electric field strength and overlap with bound biological species must be optimized to maximize the impact on the “effective refractive index” as seen by the guided optical wave. The optimization procedure is essentially the same for both sensing approaches and will be discussed based on the interferometric sensing concept.

**Figure 4.**
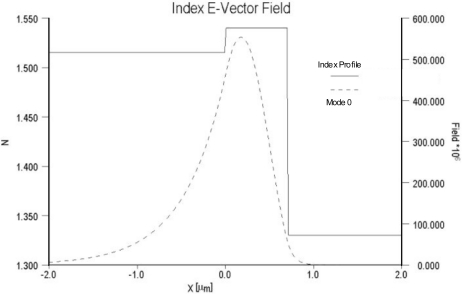
Electric field distribution for a low-contrast waveguide system.

**Figure 5.**
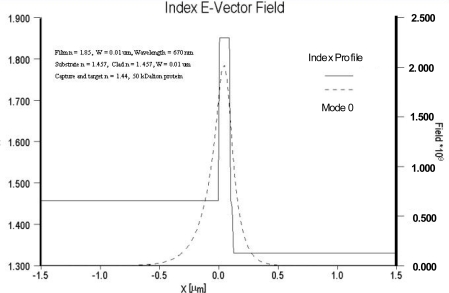
Electric field distribution of a high-contrast waveguide-system.

As noted previously, sensitivity is dependent on both the relative overlap of the evanescent field with the sensing layer as well as the electric field strength at the surface of the optical waveguide and sensing layer. For comparison purposes, the electric field distributions of two different waveguide systems are compared in Figures 4 and 5. In Figure 4 the field distribution is shown for a low contrast waveguide system typical of a glass substrate (n = 1.515) with a deposited waveguide film having a refractive index of 1.545 (Δn = 0.3) and a thickness of 0.62 μm. Figure 5 depicts the field distribution of a high contrast waveguide system with a substrate index of 1.457 and film index of 1.85. For the high contrast waveguide system, the ratio of the area under the evanescent electric field within the cover medium relative to the total area under the electric field distribution curve is noticeably larger than that of the low contrast waveguide system. Equally important, the electric field strength at waveguide surface and cover medium interface of the high contrast waveguide system is more than an order of magnitude greater than that of the low contrast system.

**Figure 6.**
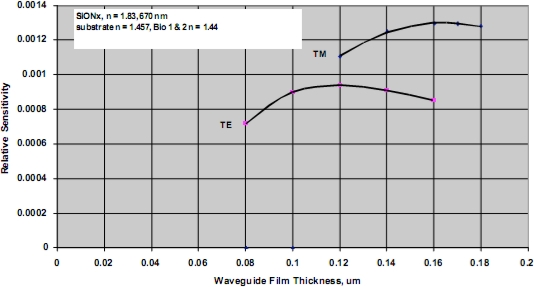
Relative Detection sensitivity for a high contrast waveguide materials system.

For a particular combination of waveguide material system, the film thickness providing maximum detection sensitivity for a biological agent can be predicted. The model used for this task assumes a waveguide with specific refractive index properties, operating at fixed optical wavelength, and a biological target with dimensions of approximately 5 nm and refractive index of 1.44 (typical for a 50 kDa protein). The analysis further assumes water as the cover medium over the waveguide surface. A typical result is shown in Figure 6, indicating optimum detection sensitivity occurs at a film thickness of approximately 0.12 μm for a silicon oxynitride waveguide film on a fused silica substrate and TE polarization. For further comparison, the TM sensitivity is also shown, and as indicated previously is only slightly better than the predicted relative sensitivity for TE polarization. The inherent advantages of TE polarization more than makes up for the slightly enhanced detection limits associated with TM polarized light. To support guiding the low contrast waveguide film must be thicker, and as a result and the low contrast system is more than an order of magnitude less sensitive than that of the high contrast system. Going to material systems with increasing substrate/film index differences yields diminishing returns as the surface interaction begins to produce excess scattering losses. Ultimately substantial loss of the guided wave intensity occurs and cross-talk between closely spaced adjacent sensing results. This phenomenon also applies to reverse symmetry waveguides, which are waveguides with a substrate index that is lower than that of the bound biological agent.

## 4. Functionalization of Waveguides for Bioassays

The sensing film at the interface between the inorganic transducer and the media to be probed is critical in biosensing applications. The overall goal is amplified signal transduction triggered by a specific recognition event between the target analyte and the capture recognition ligand. To accomplish this, research teams have prepared a variety of films with the primary goals of: (1) suppressing non-specific binding of biological molecules to the transducer surface and (2) securely attaching, usually through covalent binding, a recognition ligand. Other attributes of the sensing film, such as fluidity to provide mobility of recognition ligands for binding to multivalent proteins [14,15] are also desirable. A variety of sensing films have been utilized for fluorescence-based detection on optical waveguides—phospholipid bilayers that provide fluidity, alkyl silanes with a distal reactive group (amines, thiols, epoxides, carboxylic acids, and aldehydes), polyethylene glycol chains, poly-amino acids, and proteins adsorbed to a surface. The emphasis researchers have placed on the attributes of sensing films depends greatly on the transducer used and, consequently, how non-specific signals are addressed (through surface functionalization or through instrumentation/reference channels). For the purpose of this review, we will mention several strategies that have been explored to provide a link between biological samples and transduction, with special emphasis placed on those strategies that address both the immobilization of a recognition ligand and the prevention of non-specific binding. We will not discuss ligand immobilization specifically; this topic has been reviewed in detail before [13,16,17].

We have used supported phospholipids bilayers that contain low concentrations of small molecule receptors (e.g., GM-1) modified by the covalent conjugation of fluorescent dyes to detect cholera toxin [14,15]. Binding of multiple dye labeled GM-1 receptors to the cholera toxin brings the dyes into close proximity inducing fluorescence quenching or fluorescence resonant energy transfer. In addition to fluidity and the superior rejection of non-specific biding provided by phospholipids bilayers, multivalent biding results in enhance sensitivity by virtue of the surface avidity effect. More recently, we have used supported lipid bilayers as the sensing films for sandwich immunoassays as well.

Supported phospholipid bilayers are readily prepared on cleaned oxide surfaces by fusion of small bilayer vesicles [18–20]. Preparation of the vesicles and fusion to the surface can be accomplished in a relatively short period of time (4–6 hours) with readily available starting materials [21,22]. The resulting surface-bound bilayers can be used in sensing by attaching a capture element (such as biotin) or adding in a small amount of a functionalized lipid; the functional moiety is usually kept to a small percentage to minimize non-specific binding [22]. Several antigens have been detected using these surfaces, including *Bacillus anthracis* protective antigen (PA) [22,23], carcinoembryonic antigen (CEA) [21,24], *Mycobacterium tuberculosis* lipoarabinomannan (LAM) [25], and *Vibrio cholerae* toxin [14,15,26]. Despite the advantages of fluidity, rapid preparation and superb resistance to non-specific binding, as well as low auto-fluorescence at most useful wavelengths, phospholipid bilayers have notable disadvantages with respect to sandwich immunoassays. First, phospholipids only occur with limited functionality, the most useful being biotin. Assays that use phospholipids often rely on mobile ligands such as GM1 [26], and must be chosen carefully to select capture agents that insert into lipid bilayers. The second major disadvantage of phospholipid bilayers is instability to some complex fluids (e.g., urine) [27], detergent rinse, exposure to air, and extended periods of time in storage [27–29]. Chemical/terminal flexibility can be addressed synthetically [30]; however, the preparative route necessary to create a molecule that can interact with lipid membranes and bulk solution while simultaneously incorporating a receptor and/or a fluorescent moiety is difficult. One strategy that we explored is the synthesis of a trifunctional linker moiety that contains lipid tails (acyl chains) for membrane anchoring, a fluorescent dyes and a functional group required for attachment of the recognition ligand [30]. Stability has been addressed in a variety of ways, including chemically cross linking phospholipid bilayer [29], coating the phospholipid with a streptavidin coating [31], or capturing the bilayer in a sol-gel matrix [32]. While promising, these stabilization methods remain unproven in actual biological assays on a waveguide platform; in the case of cross linking, defects appear that actually enhance non-specific binding [29].

The use of poly- or oligoethylene glycol (PEG) chains has also been shown to reduce or eliminate non-specific binding of biomolecules to surfaces [33–38]. The main advantage of using PEG chains rather than phospholipid bilayers or other physically adsorbed polymers is the ability to conjugate them covalently to surfaces. This advantage has been demonstrated using PEG-terminated thiols on gold, and the parameters that affect the resistance of various PEG chain lengths, attachments, and terminations have also been investigated [39]. Much of the work surrounding covalently attached PEG groups, however, has not been readily applicable to dielectric substrates, which are typically composed of oxides. There have been various solutions to this problem. Laibinis *et al.* report an analogous silane that was prepared and used to coat the inner surface of a capillary for electrophoresis [40,41]. Desai and coworkers created PEG chains with terminal silanes and used these products directly to coat surfaces [42,43]. Kohler *et al.* prepared bifunctional silane-PEG conjugates to coat magnetic particles for there cell targeting application [44,45]. Other researchers have attached PEG chains [46–48] or dextran groups [49] to an amino acid backbone to effectively prevent non-specific binding. Only a few groups have prepared PEG films that simultaneously minimize non-specific interactions and maximize analyte binding for biosensing applications. For example, Zhen *et al*. [47–49] have used PEG polymers (> 3.4 kDa) functionalized with lysine groups to cover surfaces for targeted sensing applications. However, these films lack covalent attachment to the surface, which makes them unusable when extended storage and reusability are required attributes. Thompson *et al.* [50] have overcome this issue by using a silane functionalized PEG polymer (MW > 3 kDa) that is covalently attached to an oxide surface for fluorescence-based bio-recognition experiments. Further, Seidel *et al*. [51] have used patterned amine functionalized PEG polymers, which are constructed on functionalized short chain silane monolayers, for multiplexed biosensing applications. Similar work by Majumdar *et al*. used standard lithography techniques to prepare regions of functionalized silane monolayers for binding of analytes. These functional regions were surrounded by a field of PEG terminated silanes to prevent both non-specific binding to the background surface and reagent loss [52].

**Figure 7.**
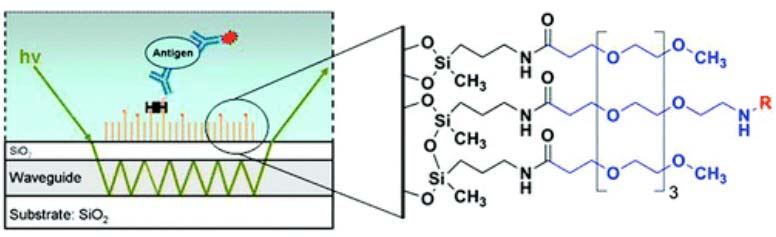
Chemistry of the Silane-based self-assembled monolayers used for waveguide functionalization (left) and a schematic representation of a waveguide-based sandwich immunoassay for biomarker detection used at LANL.

In our laboratory, we have developed silane-based self-assembled monolayers (Figure 7) that are very effective in minimization of non-specific interactions on waveguides during bioassays. For this, silica surfaces were functionalized by self-assembly of an amine-terminated silane film using both vapor- and solution-phase deposition of 3′-aminopropylmethyldiethoxysilane (APMDES). We found that vapor-phase deposition of APMDES under reduced pressure produced the highest quality monolayer films with uniform surface coverage, as determined by atomic force microscopy (AFM), ellipsometry, and contact angle measurements. The amine-terminated films were chemically modified with a mixture of carboxylic acid-terminated poly(ethylene glycol) (PEG) chains of varying functionality. A fraction of the PEG chains (0.1−10 mol%) terminated in biotin, which produced a surface with an affinity toward streptavidin. When used in sandwich immunoassays on waveguide platforms for the detection of several biomarkers such as the *Bacillus anthracis* protective antigen, these functional PEG surfaces significantly reduced nonspecific binding to the waveguide surface while allowing for highly specific binding. A great advantage of SAMs was realized in the use of these surfaces for the detection of biomarkers in urine. As mentioned earlier, urinary salts destabilize lipid bilayers resulting in high non-specific binding. However, we have been able to use SAM-functionalized waveguides for detection of urinary biomarkers with no increase in non-specific binding [27,28]. We have also successfully detected *Mycobacterium tuberculosis* virulence factors in patient urine using SAM functionalized waveguides [30]. We have found these silane-based self-assembled monolayers (SAMs) to be robust (stable for > 300 days in air), resistant to non-specific binding even with high-salt samples such as urine, resistant to washing with potent solvents, buffers and detergents and potentially reusable. The lack of fluidity of SAM surfaces precludes their use with fluroescence resonance energy transfer assays and other techniques that require mobility of the biomolecules involved. We have used planar optical waveguides functionalized with SAMs for many of our biodetection assays with excellent results (Section 6).

Two basic methods exist for preparing covalently attached PEG-terminated thin films: deposition of molecules synthesized using *de novo* preparative routes to form a self-assembled monolayer of that synthetic molecule [40–44], or stepwise functionalization of a simpler self-assembled mono-layer [27,53]. Either method is adequate for prevention of non-specific binding; however, if a biological molecule or receptor will be attached to the surface, one must weigh the pros and cons of each method. Deposition of complete molecules leads to a more efficient coating process and less ‘down time’ between assays, but requires synthetic effort to create the necessary silanes (at least two are required—one for prevention of non-specific binding (NSB), and one for ligand attachment); a “build up” method of sequential depositions and reactions on a surface slows the coating process but eliminates the synthesis. The “build up” method also requires at least two PEG reagents, but the synthetic effort required is usually reduced because many bifunctional PEG reagents are available commercially from sources such as Fluka^™^, QuantaBiodesign^™^, and Nektar^™^.

## 5. Modes of Transduction

The various modes of transduction that are fluorescence, interferometry, radiolabeling, and others, used in conjunction with waveguides and fiber optics for biodetection, have been extensively reviewed earlier [54]. This review will discuss some of the fluorescent and interferometry-based detection platforms used in conjunction with planar optical waveguides for efficient biodetection.

### 5.1. Fluorescence-based Detection of Nucleic Acids and Proteins

Fluorescence has been used for the detection of both nucleic acids and proteins on planar optical waveguides. Fluorescent labels have been used for nucleic acid detection on planar optical waveguides [55], and the technology is now available as a commercial product (Zeptosense®, Section 7). Use of fluorescent labels in the context of planar optical waveguides allows for better sensitivities and specificities, as well as ease of labeling, while detection within the evanescent field minimizes the disadvantages associated with high backgrounds associated with auto-fluorescence from complex samples. Disadvantages of fluorescence detection include the rapid photobleaching of fluorescent organic dyes conjugated to the biomolecules of interest and the potential loss of biological activity of biomolecules upon chemical conjugation of a fluorescent dye.

Wellman and Sepaniak [56] have described a magnetically assisted transport evanescent field fluoroassay (MATEFF) that takes advantage of several innovations to achieve multiplex protein detection and nucleic acid hybridization on planar optical waveguides. The investigators have demonstrated the detection of rabbit IgG and interleukin 4 in a sandwich immunoassay platform with a dynamic range of three orders of magnitude and physiologically relevant detection limits [56]. Further, they applied the technique to DNA hybridization via fluorescence emission from a DNA intercalator with excellent efficiency. Herron *et al.* have demonstrated the detection of human chorionic gonadotropin with low picomolar sensitivities on planar optical waveguides using a sandwich immunoassay platform. The reporter antibody is fluorescently labeled and the specific signal is detected using a CCD camera interface [57]. Voirin *et al.* have developed a fluorescent waveguide biosensor for the detection of antibiotic residues in milk and achieved excellent detection sensitivities [58,59]. Sol-gel silica planar optical waveguides have been doped with green fluorescent protein for development of in-line biosensors [60]. The investigators immobilized green fluorescent protein on the surface of polystyrene micro-beads and observed visual fluorescence thirty days after fabrication, allowing for real-time, in line remote biosensor network developments.

Our laboratory has developed a fluorescence-based waveguide optical biosensor and has successfully adapted the platform to the detection of biomarkers associated with several diseases such as cancer [21,24], anthrax toxins [22,23], cholera toxin [14,26], influenza [61] and tuberculosis [25]. We have been successful in developing both fluorescence resonance energy transfer and traditional fluorescence immunoassay platforms on our waveguide-biosensor. Traditionally, we have used organic dyes (AlexaFluor^™^) as the fluorescent reporter in our bioassays and obtained excellent sensitivities. However, organic dyes are easily photobleached and are not amenable to multiplexing. These issues have lead us to explore the conjugation of antibodies with photostable QDs. These conjugates have thus far found utility in multiplex detection assays for breast cancer biomarkers and anthrax lethal toxins [21,23]. The optical properties, sensitivities and assay design features of our platform are outlined in detail in Section 6 of this manuscript.

### 5.2. Interferometry-Based Detection of Biomolecules

Waveguide based optical interferometers have been used for detection of biological agents including bacteria, spores, toxins, viruses, and proteins [12,62,63]. Influenza viruses have been detected in nasal oral secretion at approximately 10^5^ plaque forming units/mL (PFU/mL) and proteins detected at concentrations of a few nanograms/mL (ng/mL). More recently nGimat^™^ has focused on detection of water born pathogens with the emphasis on enhancing detection sensitivity through improved waveguide quality and optimizing surface attachment chemistry. Using a fully integrated IOC interferometer with four interferometric sensing channels of 1.5 cm length, direct detection of *E. coli* O1:H57 has been demonstrated at concentrations in the 10^3^ cells/mL range. Interferometric approaches offer an advantage in sensitivity for the detection of intact viral particles or bacteria as the effective change in refractive index is large relative to binding events that involve much smaller biomolecules.

For these measurements, selectivity relied on antibodies covalent attached to the sensing arm of an interferometric sensing channel. The attachment process utilized clean optical chips silanized with 3, glycidoxypropyltriethoxysilane (GOPS). Oxidation of the terminal epoxide with acidic periodate, followed by reductive amination, enabled the conjugation of primary amines in the protein chains of the antibodies to the resulting surface-bound aldehydes. In all assays, the reference arm of an interferometer is coated with an antibody (IgG) that has no specificity for the analyte of interest. This coating makes the reference arm look biologically similar to the signal arm, which negates any phase changes resulting from differences in the ‘effective refractive index (n_eff_)” of the reference and signal arms.

**Figure 8.**
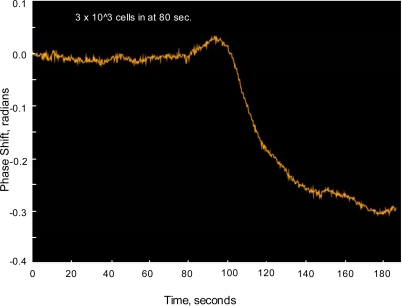
Interferometer Response with exposure to water solutions containing *E. coli* O1:H57 at 3 × 10^3^ cells/mL.

**Figure 9.**
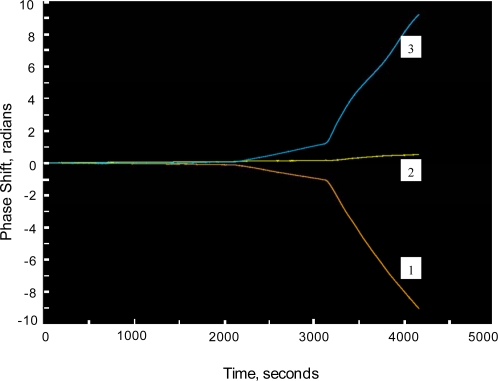
Multichannel interferometer response with exposure to water solution containing *E. coli* O1: H57 at ∼ 10^7^ cells/mL.

Figure 8 shows the phase response of a sensing channel exposed to water with the *E. coli* at a concentration of 3 × 10^3^ cells/mL. It should be emphasized that the response is due to the direct binding of the target antigen (heat killed *E. coli* O1:H57) onto the waveguide surface, that is with no labeling. The response is clearly evident within a few minutes and is typical of a diffusion limited binding process. Based on the signal-to-noise ratio, the actual detection limit is in the several hundred-cells/mL range. Figure 9 shows a multi-channel response at higher concentrations of *E. coli*. Two sensing channels, 1 and 3, utilized *E. coli* antibodies covalently attached to opposite interferometer arms, that is the sensing and reference arm orientation were reversed. This resulted in the finite fringe pattern generated at the output of each interferometer moving in opposite directions with a phase shift resulting from antigen binding to the sensing arms. The signal-processing algorithm used to deconvolve the phase signal assigned a positive or negative value to the phase shift based on direction of fringe motion. Channel 2 served as a control interferometer with both arms coated with IgG antibodies. Even though the *E. coli* concentration in the water solution is high, on the order of 10^7^ cells/mL, the control interferometer shows no response, indicating low NSB and good noise cancellation.

Microscopic examination of the sensor surface after a 10 minute exposure to aqueous sample solutions reveals that the actual surface coverage with target cells is less than 1%, implying substantial improvement in the detection process should be possible by improving overall surface coverage or binding efficiency. Limitations to overall binding efficiencies are believed to be due to diffusion limiting mechanisms, steric hindrance, poor antibody coverage, and/or denaturing.

## 6. The Los Alamos Waveguide-based Optical Biosensor

**Figure 10.**
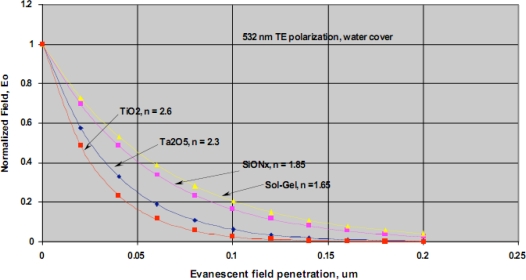
Comparison of the evanescent field decay in different waveguide platforms. Details in Section 6 of the text.

Our team has developed a waveguide-based biosensor that we have successfully adapted to the detection of biomarkers in complex biological samples. Historically, we have evaluated both sol gel and SiON_x_ waveguide materials for our sensor platform. The normalized evanescent field decay for different waveguide materials is shown in Figure 10. As indicated, there is a rapid decay in evanescent field intensity and light does not penetrate the sample beyond ∼200 nm. This spatial filtering is a distinct advantage of all evanescent wave-based detection platforms as it ensures that the bulk biological sample is not irradiated. This arrangement effectively minimizes background fluorescence and eliminates the need for extensive sample preparation when analyzing complex samples. Another critical advantage of evanescent field detection is the intense field strength at the surface where the detection occurs, facilitating observation of very low concentrations of our fluorescence reporters [22,55].

**Figure 11.**
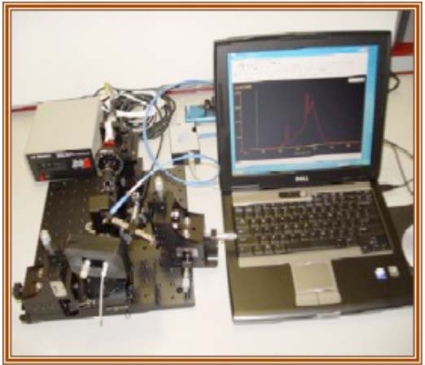
A photograph of the waveguide-based optical biosensor developed at LANL.

Much of the early work with optical waveguides in our laboratory utilized fluorescence resonance energy transfer triggered by the binding event between multivalent protein and dye-tagged receptors [14,15,64]. Sensitive detection of the cholera toxin was achieved by facilitating the binding of the toxin to its receptor (GM1) on a fluid lipid bilayer coated over waveguide surface. The toxin-binding event brought up to five GM1 and their reporter dyes conjugated to them in close proximity thereby inducing self-quenching or fluorescence resonant energy transfer and facilitating toxin detection at picomolar concentrations. Subsequently, we developed both a laboratory test-bed system and a hand-held detector for the detection of biomarkers [26]. The portable test-bed system (Figure 11) has been extensively described [21] and has been subsequently used for the detection of biomarkers associated with several diseases in a sandwich immunoassay format [21,23,24]. This system uses either a stabilized 532 nm diode pumped laser or a 635 nm diode laser for excitation, depending on the excitation wavelength of the fluorescent reporter in the assay. Grating couplers provide a facile method to couple excitation light into the thin waveguide material. Laser light is coupled into the waveguide by positioning the excitation beam onto the diffraction grating at the appropriate angle of impingement. The angle is determined by the effective mode index of the waveguiding film, film thickness, and the period and amplitude of the grating structure. These parameters are adjusted to provide a 10°–15° input coupling angle for an excitation wavelength of 532 nm. A USB 2000 fiber optic Spectrometer interface (Ocean Optics Inc. FL, USA), positioned normal to the waveguide surface to collect isotropic emissions. A long pass filter, one that does not affect transmission of light at longer wavelengths, is used to remove excitation light form the resulting fluorescence spectra. All optical components are mounted on an optical bench. A PC interface is available for monitoring and controlling of experimental parameters and to record data.

**Figure 12.**
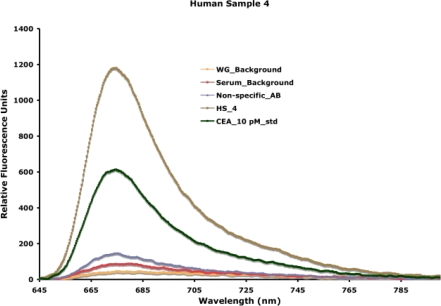
The output of a typical assay on the waveguide-based biosensor is shown. Waveguide-associated background and non-specific binding associated with control sample and the fluorescence reporter are measured in each experiment. Standard measurement of a known concentration of the biomarker of interest is then made (in this case, 10 pM of carcinoembryonic antigen, a breast cancer biomarker), followed by measurement of the biomarker concentration in an unknown patient sample (in this case, HS4). The signal measured is extrapolated against the internal standard to determine the accurate concentration of the biomarker in the patient.

Fluorescence resonance energy transfer assays, as described above, cannot be adapted to all biomolecules with excellent sensitivity and require a significant investment in ligand development. Therefore, we have concentrated on fluorescence-based sandwich immunoassays on the planar waveguide surface. We have used both phospholipid bilayers [21,23] and self-assembled monolayers (SAMs) [27,28] for waveguide functionalization, and the relative advantages and disadvantages of these techniques were discussed earlier (Section 4). Functionalized waveguides are mounted on a specially designed flow cell. A schematic representation of a fluorescence-based sandwich immunoassay on a functionalized waveguide is indicated in Figure 7 and has been described in detail before [24]. We have used this approach to detect extremely low concentrations of disease biomarkers in actual patient samples. A typical detection spectra obtained from the experimental measurement of carcinoembryonic antigen, a breast cancer biomarker, patient serum samples is indicated in Figure 12.

In all experiments, the waveguide-associated background, an intrinsic measure of impurities associated with the waveguide itself, is measured, and used as the background for data extrapolation. NSB associated with control complex samples (e.g., control serum) and the fluorescently labeled reporter antibody is measured in each experiment. As indicated in Figure 12, background fluorescence from NSB is very low, because of the detection within the evanescent field and the resistance of the functional surface (SAMs) to NSB (discussed earlier in Section 4). A capture antibody is entrapped in the surface based on biotin-avidin chemistry. Addition of the sample facilitates the binding of the antigen, when present, to the capture antibody. Addition of the reporter antibody allows for specific fluorescence measurement associated with antigen-antibody interaction. Using two antibodies, in a sandwich format, for antigen detection offers additional specificity of detection. In the detection of unknown patient samples, an internal known standard is measured first, facilitating the extrapolation of concentration of the antigen of interest in the sample. We have used this approach to detect and quantify carcinoembryonic antigen in serum and nipple aspirate fluid from patients with abnormal mammograms [24] and obtained 100% corroboration with disease progression in a blind study. The limit of detection of our assay is 500 femtomolar (< 1 ng/mL), which is lower than that achieved by traditional plate-based assay formats. Similarly, we have been able to detect *Mycobacterium tuberculosis* specific virulence factors in urine from infected patients, also in a blind study [25]. Our team has also synthesized carbohydrate ligands for the discriminatory detection of influenza viruses using a convergent strategy and demonstrated the detection of intact viruses on our waveguide-based platform [61]. More recently, we have successfully conjugated antibodies with QDs and used them in a multiplex detection assay for *Bacillus anthracis* lethal factor and protective antigen. We were able to detect these antigens with much better sensitivity than attained by conventional plate-based immunoassays using the same antibodies [21–25,65(unpublished data)].

## 7. Commercial Technologies Based on Optical Waveguides:

Many of the bioassay technologies using planar optical waveguides have been commercialized. Discussion of all them is beyond the scope of this review. To educate the reader on the variety of bio-applications using waveguide technologies, we will discuss four frequently used detection platforms in some detail. A summary of some technologies currently under development and others (discussed in greater detail below) that are already commercial products is presented in Table 2. This is by no means an exhaustive list of planar optical waveguide-based biosensing technologies but an attempt to capture the diversity of analytes and measurement strategies that are amenable to waveguide-based detection. As indicated in the table, waveguides have been used for detection of proteins, carbohydrates, DNA, intact viruses and several other analytes of interest. Not all information is readily available for all technologies, which accounts for some of the gaps in the Table 2.

### 7.1. Corning EPIC^®^ System

The Corning EPIC^®^ system is a label-free plate-based technology, where resonant waveguide grating sensors are coated on each well of a 384 well plate that has been used since 2005. The sensors are coated with a surface chemistry that facilitates the covalent attachment of biomolecules *via* primary amines. Binding of the target antigen to the immobilized capture molecules on the sensor results in a change in the local index of refraction, triggering a shift in the wavelength of light reflected from the sensor. The Epic^®^ system can be used with various high throughput screening instrumentation, and can read up to 40,000 wells in 8 hours. The technology has been adapted to detection of specific proteins, biochemical assays, cell-based assays and fibronectin measurements, to name a few [69,70]. The need for individual optimization, baseline measurements and the non-specific binding issues associated with complex biological samples need to be addressed for this technology in greater detail.

### 7.2. SRU Biosystems BIND^™^

The Biomolecular Interaction Detection (BIND^™^) system for label-free measurements of interactions between biological molecules or cells, launched in 2005, are also comprised of optical gratings coated on a microtitre plate, similar to the Corning system described in Section 7a. Illumination of the gratings with a broadband white light source resonantly coupled to the sensor surface results in reflection of a single wavelength of light (Peak Wavelength Value, PWV), with an increase in intensity when a biomolecules binds to the sensor [71]. The shift in PWV is proportional to mass density of material bound to the sensor surface and can be measured real time. The BIND^™^ Biosensor microplates are available in 96-, 384-, 1536-well formats for several assay format including, cell based assays, small molecule assays and antibody screening. Again, large assay volume and the need for custom immobilized plate’s limits application of this technology.

### 7.3. Zeptosense™ Assay

Zeptosense™, launched in 2004, is based on planer waveguide technology and includes a fluorescence reader and micro array chips with integrated microfluidics to perform multiplexed, quantitative bimolecular interaction analysis even in complex samples [72]. The planar waveguide chips are manufactured on a transparent glass or plastic substrate, coated on one side with a 150–200 nm higher refractive index light guiding layer. Light is coupled into the waveguide *via* a diffractive grating creating an evanescent field, which extends approximately 100 nm into the solution on the chip. Illumination is thus confined to the fluorophores on the surface, and readout is obtained through a 12-bit CCD camera. The ZeptoREADER™ is a fluorescence based microarray readout system (532 and 635 nm lasers), which utilizes ZeptoCHIPs™ optimized for DNA and protein arrays.

### 7.4. Microvacuum OWLS 120™

OWLS 120™ is a label free biosensor that utilizes optical waveguide light mode spectroscopy (OWLS) that have been used since 2005. The system measures refractive index changes with antigen binding and can be used for the investigation of absorption and binding processes in real time [73]. The light mode spectrum is obtained by varying the angle of incidence of the laser light and from this measurement the density of the absorbed material can be determined.

### 7.5. mBio Diagnostics

mBio Diagnostics, a division of Precision Photonics Corporation, is developing low-cost medical diagnostics focusing on technologies to move from central testing laboratories to clinics. The system utilizes integrated planar waveguides and a flow cell system, which facilitates high sensitivity fluorescence measurements, and have currently adapted it for serological detection of HIV infection in serum [53]. Some of their sensor devices were launched in 2008.

## 8. Summary and Conclusions

Waveguides have extensively been used for bio-sensing applications. Detection within the evanescent field allows for enhanced sensitivity of detection, while minimizing non-specific interactions associated with auto fluorescence in complex biological samples such as serum. Adaptation of waveguides to the detection of proteins and nucleic acids has also facilitated the furthering of ancillary technology such as functional surfaces and ligands, which benefit other research areas as well. This review is a comprehensive summary of the physical and optical properties of planar optical waveguides and elaborates on waveguide-functionalization for use in biodetection assays. We have attempted to summarize the current literature on fluorescence and interferometric detection using planar optical waveguides, highlighting both research and commercialization efforts in this field. The review is comprehensive, but not complete, because of the extensive research effort in this area. Several research applications such as silicon photonics biosensors have not been discussed here, although they have been reviewed elsewhere [74]. It is interesting to note that certain technologies such as porous silicon-based devices that suffer from several issues for use in monolithic or hybrid optoelectronic integration devices have excelled in biological and chemical sensing applications, because of the same features.

## Figures and Tables

**Table 1.** Thin film deposition techniques.

**Method**	**Advantages**	**Disadvantages**

Electron beam	Versatility - materials	Film density, uniformity
Low pressure chemical vapor deposition, LPCVD	Film density, substrate numbers	High temperatures, slow
Magnetron Sputtering	Surface coverage, density	High scatter, high scattering losses, material cost
Ion beam sputtering	Low optical losses, film density, film adhesion	Slow deposition rates.

**Table 2.** Summary of select technologies, both research and commercial, that use Single Mode Planar Optical Waveguides.

	***Research Technologies***				

	***Analyte/Target***	***Waveguide***	***Functionalization***	***Measurement***	***Reference***

**1**	Sucrose Limit of detection 2.5 × 10^−11^ M	Sol-gel	Agarose, guar-gum	Enzymatic reaction	[7]
**2**	Serine-threonine protease	Ta_2_O_5_	APDMES Silanization	ATR photometry	[66]
**3**	DNA Hybridization	Ta_2_O_5_	GOPTS silanization	Fluorescence	[67]
**4**	Intact Influenza Virus hemagglutinin 0.0005 HA units/mL	Si_3_N_4_	Silanization	Interferometry	[62,68]
**5**	Intact viruses (10^6^ particles/mL influenza), protein (sub- to low picomolar) and carbohydrate (low picomolar) biomarkers	Si_3_N_4_	Silanization, self assembled monolayers, phospholipids bilayers	Fluorescence	[22–24,61]

	***Commercial Technologies***				

	***Company***	***Waveguide***	***Applications***	***Measurement***	***Reference***

**1**	Corning EPIC®	Resonant waveguide grating	Enzyme, small molecule, protein and DNA detection, antibody profiles and cell based studies	Refractive index	[69,70]
**2**	SRU BIND®	Nanostructured optical grating	Detection of antigen binding to immobilized antibodies, hybridoma screening, antibody ranking, protein and cell assays	Refractive index	[71]
**3**	Zeptosense™	Planar waveguide	Protein detection in cultured cells, tissue and depleted serum, DNA	Fluorescence	[72]
**4**	Microvacuum OWLS™	Planar waveguides	Protein adsorption, antigen antibody binding, drug screening, protein-lipid bilayer interactions	Refractive index	[73]
